# Association of T‐Cell Profiles With Disease Severity, Drug‐Induced Liver Injury, and Treatment Completion in Tuberculosis

**DOI:** 10.1111/crj.70114

**Published:** 2025-08-01

**Authors:** Yifan He, Xubin Zheng, Zihan Dang, Xiaohui Hao, Yidian Liu, Peng Wang, Yingying Chen, Ying Wang, Wei Sha

**Affiliations:** ^1^ Clinic and Research Center of Tuberculosis, Shanghai Key Laboratory of Tuberculosis Shanghai Pulmonary Hospital, School of Medicine, Tongji University Shanghai China; ^2^ Department of Health Studies and Applied Educational Psychology Columbia University New York New York USA; ^3^ Department of Microbiology and Immunology Institute of Immunology, Shanghai Jiao Tong University School of Medicine Shanghai China

**Keywords:** drug‐induced liver injury, immune checkpoint molecules, T‐cell phenotypes, treatment response, tuberculosis

## Abstract

**Background:**

Tuberculosis (TB) treatment is challenged by a long duration, poor adherence, and the high risk of drug‐induced liver injury (DILI). T‐cell immunity is essential for anti‐mycobacterial defense, but current immune‐monitoring methods poorly reflect disease severity and treatment response. Correlations of immune subpopulations with TB severity, DILI, and treatment prognosis remain poorly understood.

**Methods:**

Peripheral blood mononuclear cells were collected from confirmed TB patients (*n* = 40). Multiparameter flow cytometry analysis was used to assess previously defined TB‐associated T‐cell phenotypes based on the co‐expression of cytokines and immune checkpoint molecules following stimulation with two *Mycobacterium tuberculosis* peptides: culture filtrate protein 10 and early secreted antigenic target 6. Patients were subgrouped by disease severity, DILI, and treatment regimen (16‐week short course vs. 24‐week standard).

**Results:**

Specific subsets (14/124) were found to be associated with disease severity. Notably, six of 14 subsets were positive for programmed death‐ligand 1 (PD‐L1), indicating its potential role in disease progression. DILI was associated with three interleukin (IL)‐21^+^ subsets (naïve CD4^+^, memory CD8^+^, and interferon [IFN]‐γ^−^ CD4^+^ T cells) and IL‐17^+^ memory CD8^+^ T cells, along with PD‐L1^+^TIM‐3^+^CD4^+^ T cells (all *p* < 0.05). The 16‐week and 24‐week treatment groups showed a significant difference in IFN‐γ^+^ naïve CD8^+^ T cells at week 16 (*p* = 0.013), but not at treatment completion (*p* = 0.393), despite the different durations.

**Conclusions:**

This study identifies specific T‐cell phenotypes associated with TB severity, DILI, and treatment dynamics, highlighting potential immune markers for disease monitoring and DILI prediction.

## Graphical abstract

Fourteen T cell phenotypes were associated with disease severity, including the number of pulmonary cavities. Five T cell phenotypes were associated with drug‐induced liver injury (DILI). The 4‐month and 6‐month treatment groups showed a significant difference in IFN‐γ^+^ naïve CD8^+^ T cells at week 16 but not at treatment completion.

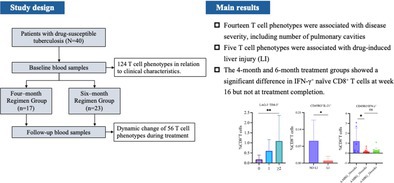

## 1 Introduction

Tuberculosis (TB) remains a significant global health issue, particularly in developing countries [1]. Current TB management faces two main challenges: Traditional methods [2] (e.g., culture conversion and sputum smear microscopy) are hindered by delayed results and low sensitivity [3], while advanced techniques (e.g., GeneXpert MTB/RIF) detect active pulmonary TB but are ineffective for latent and extrapulmonary TB [4, 5]. Additionally, drug‐induced liver injury (DILI) caused by anti‐TB regimens occurs in 2%–28% of patients [6], severely affecting treatment compliance and outcomes. New approaches focused on the host biomarkers involved in host immune responses could offer improved diagnosis, DILI risk prediction, and treatment monitoring [7].

TB pathogenesis is deeply intertwined with T‐cell immunity. CD4^+^ and CD8^+^ T cells mediate anti‐TB immunity through cytokine secretion and immune checkpoint regulation. Patients with active TB typically exhibit T‐cell exhaustion (e.g., elevated programmed death‐1 [PD‐1] expression) and inflammatory dysregulation (e.g., interleukin [IL]‐6/tumor necrosis factor [TNF]‐α imbalance), with immune signatures that closely correlate with bacterial burden and lung damage [8, 9]. While traditional immunological assays struggle to resolve the functional heterogeneity of T‐cell subsets at single‐cell resolution, flow cytometry enables high‐throughput quantification of cytokine profiles and checkpoint markers on CD4^+^/CD8^+^ T cells. This technology has already demonstrated utility in TB research. For instance, measurement of CD38^+^CD27^−^CD4^+^ T‐cell frequencies can differentiate active TB from latent infection, while monitoring activation markers predicts treatment response [8, 9]. However, the integration of flow cytometry with clinical phenotypes (e.g., disease severity, DILI) and treatment dynamics remains underexplored, highlighting a critical research gap.

Recent advances in immune profiling, including high‐throughput single‐cell sequencing and multiparametric flow cytometry [10], have enabled the systematic characterization of host immune responses in TB. These technologies have identified specific immune cell subsets (e.g., PD‐1^+^CD4^+^ T cells, and myeloid‐derived suppressor cells) associated with disease severity and treatment response, offering potential biomarkers for noninvasive diagnosis and dynamic immune monitoring. Harnessing comprehensive immune profiling represents a promising strategy to enhance TB management, ultimately improving treatment outcomes.

Given the limitations of current TB diagnostics and recent progress in immunomics, we employed flow cytometry to systematically profile the expression of cytokines (IFN‐γ, TNF‐α, IL‐2, IL‐10, IL‐17, IL‐21) and immune checkpoint markers (lymphocyte activation gene 3 [LAG‐3], T‐cell immunoglobulin and mucin domain‐containing 3 [TIM‐3], PD‐1, programmed death‐ligand 1 [PD‐L1], and cytotoxic T‐lymphocyte–associated protein 4 [CTLA‐4]) on naïve/memory CD4^+^/CD8^+^ T cells from TB patients. The main objectives were to (1) define correlations between immune markers and disease severity, (2) identify DILI‐predictive immune signatures, and (3) develop immune‐based models for treatment endpoints. By mapping immune–clinical associations, this work aims to advance precision strategies for TB management.

## 2 Methods

### 2.1 Sample Collection and Flow Cytometric Profiling of T‐Cell Subsets

All sample preparation and handling steps were conducted in a designated biosafety hood, following standard protocols for unfixed human blood samples. Blood was collected at enrollment (baseline), after 4 weeks, and at the end of treatment (16 or 24 weeks for the 4‐month regimen [4‐MRG] and 6‐month regimen [6‐MRG] groups, respectively). Density gradient centrifugation was used to isolate peripheral blood mononuclear cells (PBMCs) from blood collected into K3‐EDTA tubes. PBMCs were stimulated with *Mycobacterium tuberculosis* (MTB) peptide pools of culture filtrate protein 10 (CFP‐10) and early secreted antigenic target 6 (ESAT‐6) (Sangon, Shanghai, China) in a 200‐μL volume in 96‐well U‐bottom plates (Corning, NY, USA) in a 5% CO_2_ incubator at 37°C for 14 h. GolgiStop (BD Biosciences, Franklin Lakes, NJ, USA) was then added, and the PBMCs were incubated for a further 6 h under the same conditions. A 6‐h stimulation with phorbol 12‐myristate 13‐acetate (PMA) plus ionomycin (Sigma, St. Louis, MO, USA) mixed with GolgiStop served as the positive control, while culture medium only was employed as the negative control.

Employing high‐dimensional flow cytometry, we assessed the expression of the following surface markers using the corresponding antihuman antibodies: CD3‐BV510, CD8‐PE‐Cy7, CD45RO‐FITC, CD27‐APC‐H7, PD‐1‐PE, PD‐L1‐BV421, CTLA‐4‐BV786, TIM‐3‐BV711, LAG‐3‐APC‐R700, IFN‐γ‐PERCP‐CY5.5, IL‐2‐BV421, IL‐10‐PE, IL‐21‐AF647, TNF‐α‐BV650, and IL‐17A‐BV786. Surface marker‐labeled cells were fixed with Cytofix/Cytoperm solution (BD Biosciences) for 20 min and then labeled with antibodies for 40 min in the dark at 4°C. Labeled cells were acquired using a BD LSRFortessa Flow Cytometer (BD Biosciences), and data were analyzed with FlowJo software v10 (FlowJo LLC, BD Biosciences). Doublets were excluded by forward scatter (FSC)‐A versus FSC‐H gating.

At baseline, we analyzed 124 immune subpopulations in each patient. Specifically, we focused on the proportions of memory (CD45RO^+^) and naïve (CD45RO^−^) subsets of CD4^+^ and CD8^+^ T cells that secreted cytokines IFN‐γ, TNF‐α, IL‐2, IL‐10, IL‐17, and IL‐21. Additionally, we analyzed pairwise combinations of any two among IFN‐γ, TNF‐α, IL‐2, IL‐10, IL‐17, and IL‐21, as well as the immune checkpoint molecules lymphocyte activation gene 3 (LAG‐3), T‐cell immunoglobulin and mucin domain‐containing 3 (TIM‐3), PD‐1, programmed death‐ligand 1 (PD‐L1), and cytotoxic T‐lymphocyte‐associated protein 4 (CTLA‐4) and determined their proportions within these subpopulations.

At the follow‐up time points, we assessed treatment effects by monitoring 56 cell subsets, including baseline cytokine‐secreting profiles, pairwise combinations, and the tripartite IFN‐γ/IL‐2/TNF‐α expression. Immunophenotypes were compared between treatment groups at week 16 and their respective endpoints (16 weeks for 4‐MRG; 24 weeks for 6‐MRG).

### 2.2 Consent

Written informed consent was obtained from all participants in the parent study. Detailed information regarding the study design, randomization, and treatment allocation of the parent study was previously published [11].

### 2.3 Study Participants

All newly diagnosed, first‐line drug‐sensitive pulmonary TB patients (male and female) who enrolled under the parent study and started treatment between June 2018 and July 2019 were included, with diagnosis confirmed by sputum culture and radiological findings.

Patients were excluded if they had HIV infection, chronic obstructive pulmonary disease (COPD), type 2 diabetes mellitus, chronic viral hepatitis, chronic kidney disease, rheumatoid arthritis, or other chronic illnesses. Comorbidities were diagnosed based on a combination of medical history, physical examination, and laboratory findings.

### 2.4 Treatment Regimens and Monitoring

Two distinct treatment regimens were implemented for the participants: (1) a standard 6‐month regimen, in adherence to the World Health Organization treatment guidelines, comprising 2 months of intensive treatment with isoniazid (H), rifampicin (R), pyrazinamide (Z), and ethambutol (E), followed by 4 months of continuation treatment with H and R (2HRZE/4HR) and (2) a 4‐month short‐course therapy [11] comprising 2 months of treatment with H, prothionamide (P), moxifloxacin (M), and Z and another 2 months of treatment with H, P, and M (2HPMZ/2HPM) (Table S1).

Both groups were monitored at treatment initiation (baseline) and at 4 weeks post initiation. The 4‐MRG group had an additional follow‐up at their treatment endpoint (16 weeks), while the 6‐MRG group was assessed at both 16 weeks and their treatment endpoint (24 weeks).

### 2.5 Information Collection and Variable Definitions

Smear grade [12, 13], lung involvement (≤ 3 lobes or > 3 lobes on computed tomography [CT] scanning), and the number of pulmonary cavities (0, 1, or ≥ 2) were considered clinical indicators of active TB (ATB). Smear grade was classified on the basis of culture results, following the 2021 Chinese National Guidelines: negative (−), scanty, 1+, 2+, and 3+.

To assess DILI during treatment, liver biochemistry had to meet at least one of the following criteria for acute DILI: (1) alanine aminotransferase (ALT) ≥ 5 times the upper limit of normal (ULN), (2) alkaline phosphatase (ALP) ≥ 2 × ULN (particularly when accompanied by an elevated γ‐glutamyl transferase level with bone disease excluded), or (3) ALT ≥ 3 × ULN and total bilirubin (TBil) ≥ 2 × ULN [14].

To evaluate disease prognosis, we monitored the time for conversion of sputum cultures to a negative status within a 3‐month treatment period and classified the patients into three groups accordingly: < 1 month, < 2 months, and ≥ 3 months. This classification was used to analyze the relationship between baseline CD4^+^ and CD8^+^ T‐cell phenotypes and disease prognosis.

### 2.6 Statistical Analysis

Baseline data are presented as follows: proportions for categorical variables, means and standard deviations (SDs) for normally distributed continuous variables, and medians with interquartile ranges (IQRs) for nonnormally distributed continuous variables. The Kruskal–Wallis and Mann–Whitney tests were employed to compare baseline clinical indicators of disease severity. At follow‐up, we used the Mann–Whitney *U* test to compare the expression of 56 immune cell subsets between the two groups at week 16 and at treatment completion (16 weeks for 4‐MRG and 24 weeks for 6‐MRG). When comparisons involved more than two groups, the false discovery rate (FDR) method was applied to correct for multiple testing and control the expected proportion of false positives. All *p‐*values were interpreted in the context of multiple comparisons, with a *p*‐value < 0.05 considered potentially significant. Statistical analyses were performed and graphs were created using Prism 9.4.1 software (Graph Pad Software Inc., La Jolla, CA, USA).

## 3 Results

### 3.1 Demographics and Clinical Characteristics of the Study Population

**FIGURE 1:**
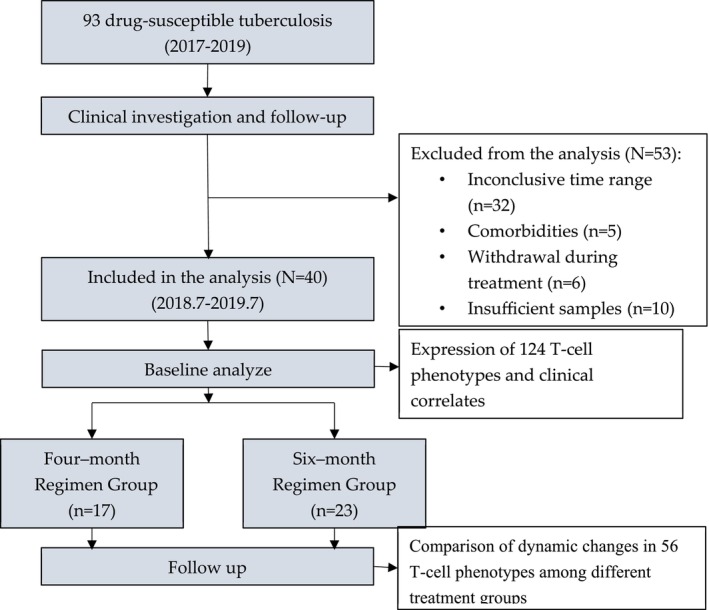
Flowchart of the enrollment for study participants. Patients with drug‐susceptible tuberculosis and available serum samples from a parent clinical trial were included.

After excluding 53 patients from the parent study who failed to meet the eligibility requirements, a total of 40 patients (17 in the 4‐MRG group and 23 in the 6‐MRG group) fulfilled the criteria for serum analysis (Figure 1) [15]. The enrolled cohort had a median age of 37 years, and the majority were men (72.5%). Baseline demographic and clinical characteristics were comparable between the two treatment groups, as determined by standardized data collection and analysis methods (Table 1).

**TABLE 1:** Demographic and clinical characteristics for study patients at baseline.

Characteristics	In total (*n* = 40)	Treatment regimen		*p*‐value
		4‐month (*n* = 17)	6‐month (*n* = 23)	
Age, years^a^	37 (28.0, 53.0)	31.0 (26.0, 48.0)	38.0 (30.0, 59.0)	0.17
Male	29 (72.5)	15 (88.2)	14 (60.9)	0.08
Body mass index^a^	19.8 (18.5, 22.3)	21.6 (18.8, 22.6)	19.5 (17.6, 20.3)	0.18
No. of infected pulmonary zones^a^	4 (3, 6)	3 (2.5, 5)	4 (3, 6)	0.21
No. of pulmonary cavities				0.50
0	12 (30.0)	6 (35.3)	6 (26.1)	
1	13 (32.5)	6 (35.3)	7 (30.4)	
≥ 2	15 (37.5)	5 (29.4)	10 (43.5)	
Smear grade				0.35
Negative	1 (2.5)	0 (0.0)	1 (4.4)	
Scanty and 1+	14 (35.0)	5 (29.4)	9 (39.1)	
2+	13 (32.5)	6 (35.3)	7 (30.4)	
3+ and 4+	12 (30.0)	6 (35.3)	6 (26.1)	
Time to sputum conversion, month				0.34
≤ 1	24 (60.0)	9 (52.9)	15 (65.2)	
2	12 (30.0)	5 (29.4)	7 (30.4)	
≥ 3	4 (10.0)	3 (17.6)	1 (4.3)	
Interferon gamma release assays				> 0.99
Positive	34 (85.0)	13 (76.4)	21 (91.4)	
Negative	3 (7.5)	2 (11.8)	1 (4.3)	
Unknown	3 (7.5)	2 (11.8)	1 (4.3)	
Liver injury during treatment				0.14
No	31 (77.5)	11 (64.7)	20 (87.0)	
Yes	9 (22.5)	6 (35.3)	3 (13.0)	

*Note:* Data were presented as the number (percentage) otherwise specified.

^a^ Median (interquartile range).

### 3.2 Correlation Between Baseline CD4^+^and CD8^+^ T‐Cell Phenotypes and Indicators of Clinical Severity

**FIGURE 2:**
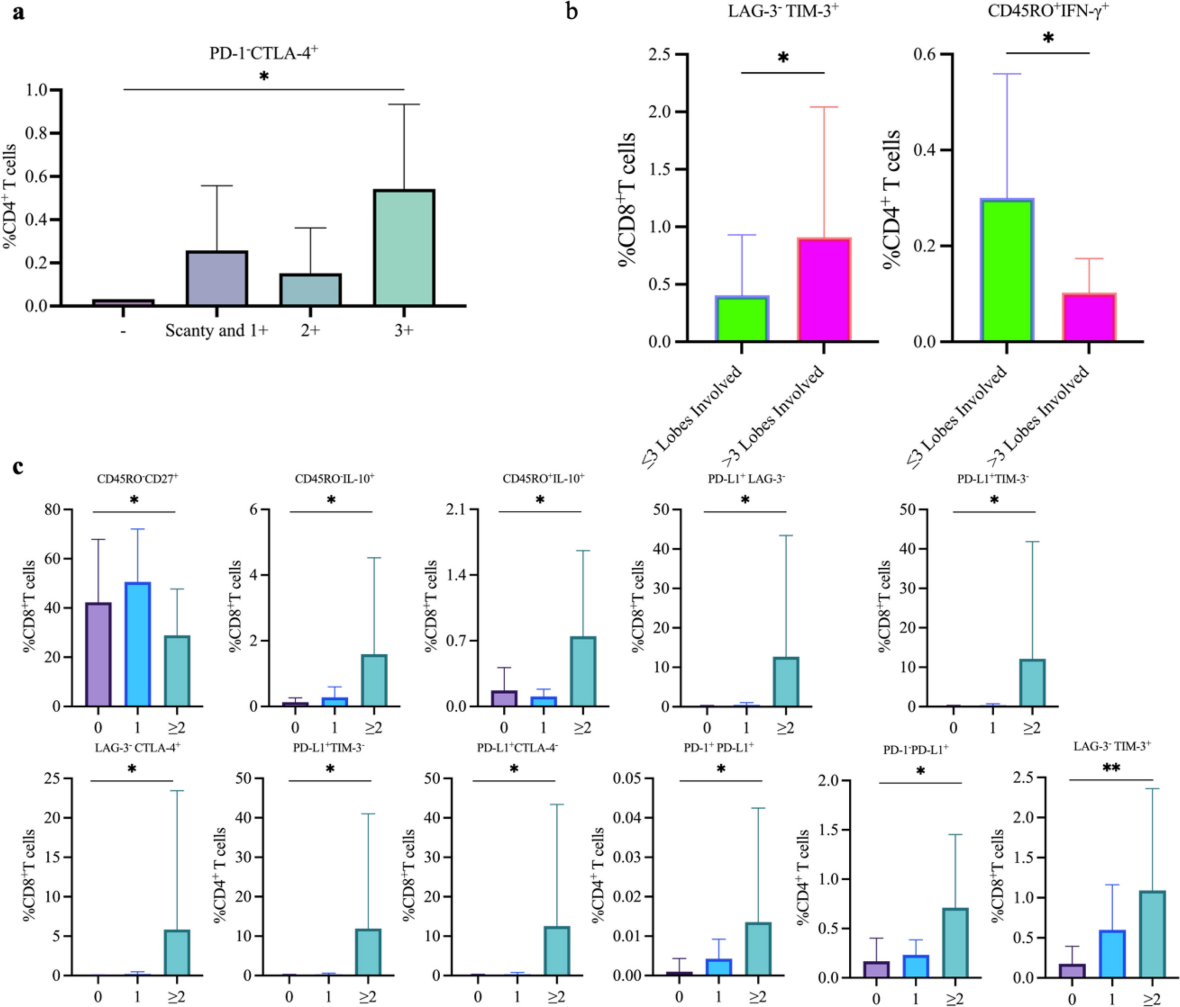
Significant intergroup variations in CD4^+^ and CD8^+^ T‐cell phenotypes among tuberculosis patients stratified by clinical characteristics. Proportions of CD4^+^ and CD8^+^ T‐cell phenotypes between patient subgroups stratified by: sputum smear positivity, categorized by bacterial load as negative (−), scantly positive and 1+, or 2+ (a); number of lung lobes involved (≤ 3 or > 3) (b); and number of cavities (0, 1, or ≥ 2) (c). Data analyzed by Kruskal–Wallis test (a, c) or two‐tailed Mann–Whitney test (b); *p < 0.05, **p < 0.01. CTLA‐4: cytotoxic T‐lymphocyte–associated protein 4; IL: interleukin; IFN: interferon; LAG‐3: lymphocyte activation gene 3; PD‐1: programmed death‐1; PD‐L1: programmed death‐ligand 1; TIM‐3: T‐cell immunoglobulin and mucin domain‐containing 3.

First, we compared the profiles of CD4^+^ and CD8^+^ T cells across patients stratified by clinical severity, as indicated by sputum smear grade, lung involvement, and number of cavities. Among these parameters, only smear grade was significantly associated with the immune phenotype: The proportion of PD‐1‐CTLA‐4^+^CD4^+^ T cells differed significantly across smear grades (*p =* 0.016*)*, while no other phenotypes showed significant associations. Figure 2a shows an upward trend in this population with increasing smear grade, suggesting a link to bacterial load in the sputum. Patients with involvement of > 3 lung lobes showed a higher proportion of LAG‐3^−^TIM‐3^+^ CD8^+^ T cells (*p* = 0.044) and a lower proportion of IFN‐γ^+^ memory CD4^+^ T cells (*p* = 0.032) compared with those with ≤ 3 lobes affected (Figure 2b). Lung involvement, assessed by the proportion of lesion area on CT imaging, indicated that these T‐cell phenotypes may be associated with the extent of pulmonary damage. Patients stratified into three groups by the number of cavities (0, 1, and ≥ 2) also showed significant differences in T‐cell phenotypes. Among CD8^+^ T cells, we observed differences in the proportions of CD45RO^−^CD27^+^ (*p* = 0.019), CD45RO^−/+^IL‐10^+^ (*p* = 0.024)/(*p* = 0.042), LAG‐3^+^TIM‐3^+^ (*p* = 0.016), LAG‐3^‐^CTLA‐4^+^ (*p* = 0.016), PD‐L1^+^TIM‐3^−^ (*p* = 0.038), and PD‐L1^+^LAG‐3^−^ (*p* = 0.019) subsets. For CD4^+^ T cells, differences were found in PD‐L1^+^CTLA‐4^−^ (*p* = 0.039), PD‐L1^+^TIM‐3^−^ (*p* = 0.039), PD‐1^+^PD‐L1^+^(*p* = 0.049), and PD‐1^−^PD‐L1^+^(*p* = 0.028) populations (Figure 2c).

Among the 60 pairwise combinations of inhibitory receptors (LAG‐3, TIM‐3, CTLA‐4, PD‐1, PD‐L1), eight combinations showed significant differences, of which six were PD‐L1^+^. This result indicated that PD‐L1 may play an important role in T‐cell immune regulation and disease progression. Notably, except for the proportion of CD45RO^−^CD27^+^ CD8^+^ T cells, all other T‐cell phenotypes were negatively correlated with the severity of the disease. Thus, the increasing proportions of these T‐cell phenotypes with greater disease severity suggest their potential as biomarkers of disease burden.

### 3.3 Baseline CD4^+^and CD8^+^ T‐Cell Phenotypes and Disease Prognosis

**FIGURE 3:**
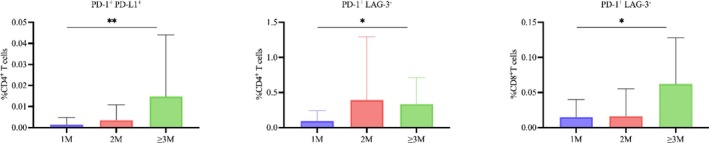
Impact of sputum smear conversion time on the proportions of CD4^+^ and CD8^+^ T‐cell phenotypes in tuberculosis patients. Proportions of the indicated T‐cell populations among three groups of patients stratified by time to sputum smear conversion: within 1 month (1 M), within 2 months (2 M), and ≥ 3 months (≥ 3 M). Data are presented as means ± standard deviation (SD; vertical error bars), analyzed by Kruskal–Wallis test; *p < 0.05, **p < 0.01. LAG‐3: lymphocyte activation gene 3; PD‐1: programmed death‐1; PD‐L1: programmed death‐ligand 1.

Based on sputum culture conversion to a negative status within a 3‐month treatment period, patients were classified into three groups: conversion within 1 month, within 2 months, and at 3 months or later. Univariate analysis of 124 combinatorial T‐cell phenotypes identified three subsets significantly associated with the duration of smear conversion (*p <* 0.05): PD‐L1^+^LAG‐3^−^ CD4^+^ (*p* = 0.038) and CD8^+^ (*p* = 0.047) T cells, ^+^PD‐L1^+^ CD4^+^ T cells (*p =* 0.008) (Figure 3). Notably, all three cell subsets were characterized by PD‐L1 expression.

### 3.4 Baseline CD4^+^ and CD8^+^ T‐Cell Phenotypes and Posttreatment Liver Injury

**FIGURE 4:**
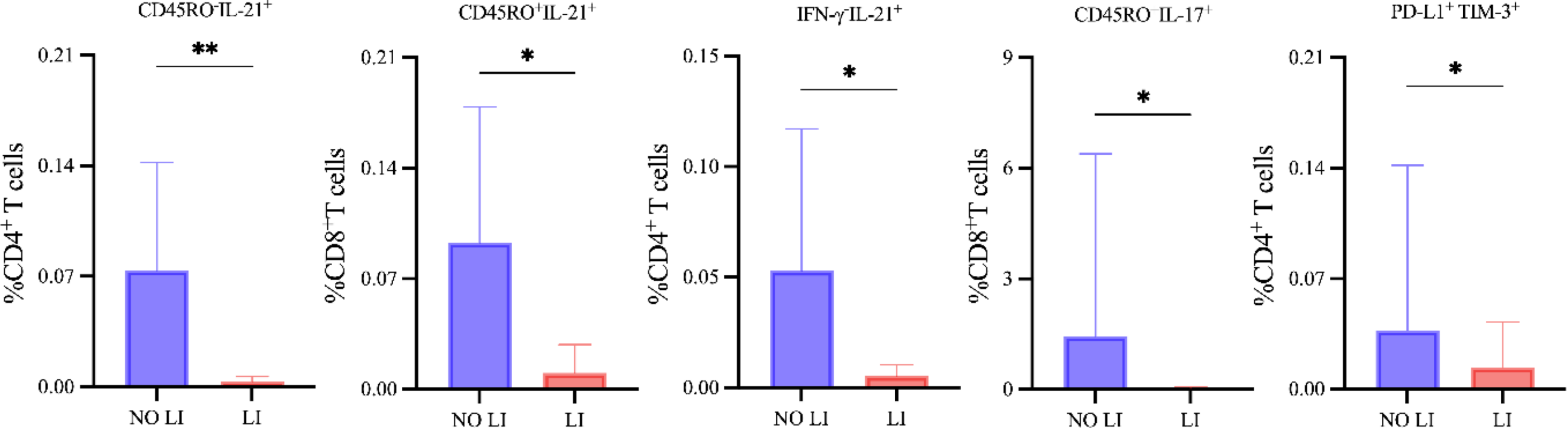
Effect of drug‐induced liver injury (DILI) on the proportions of CD4^+^ and CD8^+^ T‐cell phenotypes in tuberculosis patients. Proportions of the indicated T‐cell populations among two groups of patients stratified by no DILI (NO LI) and DILI (LI). Data analyzed by two tailed Mann–Whitney test; *p < 0.05, **p < 0.01. IL: interleukin; IFN: interferon; PD‐L1: programmed death‐ligand 1; TIM‐3: T‐cell immunoglobulin and mucin domain‐containing 3.

Among the 40 enrolled patients, nine (22.5%) experienced DILI during treatment, while the remaining 31 (77.5%) showed no evidence of liver injury (Table 1). DILI was associated with higher frequencies ^+^of IL‐21 naïve CD4^+^ T cells, IL‐21^+^ memory CD8^+^ T cells, IL‐21^+^IFN‐γ^−^ CD4^+^ T cells, IL‐17^+^ memory CD8^+^ T cells, and PD‐L1^+^TIM‐3^+^ CD4^+^ T cells (all *p <* 0.05) (Figure 4).

### 3.5 Cytokine Dynamics During TB Treatment in Active TB Infection

**FIGURE 5:**
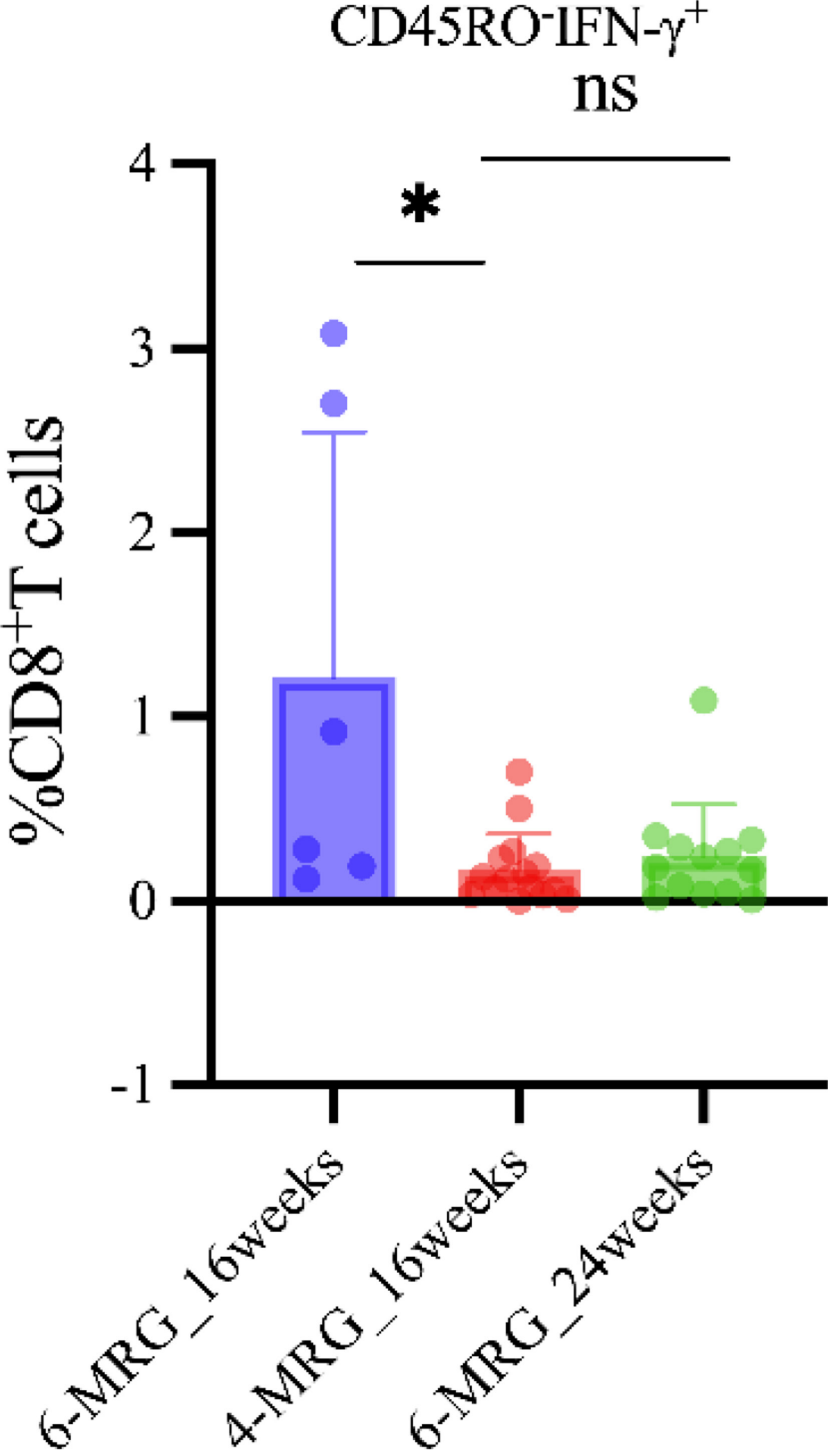
Impact of treatment regimen and duration on the CD45RO^−^IFN‐γ^+^CD8^+^ T‐cell phenotype in tuberculosis patients. Proportions of CD45RO^−^IFN‐γ^+^ CD8^+^ T‐cells across three groups: 6‐MRG_16weeks (16‐week standard regimen), 4‐MRG_16weeks (16‐week shortened regimen), and 6‐MRG_24weeks (24‐week standard regimen). Data presented as individual values (dots) with group means (horizontal lines) and standard deviations (SDs, error bars), analyzed by two‐tailed Mann–Whitney tests between the 16‐week standard and shortened regimens, and the 16‐week and 24‐week regimens; *p < 0.05. Horizontal lines and error bars denote central tendency and variability, respectively. IFN: interferon; ns: not significant (*p* > 0.05).

Next, we compared the T‐cell immunophenotypes of patients at two time points: week 16 (treatment endpoint for 4‐MRG and intermediate time point for 6‐MRG) and treatment endpoint (week 16 for 4‐MRG and week 24 for 6‐MRG). At 16 weeks of treatment, the proportion of CD45RO‐IFN‐γ^+^ CD8^+^ cells in the short‐course 4‐MRG group was significantly higher than that in the standard 6‐MRG group (*p* = 0.036) (Figure 5). However, when the two groups were compared for this indicator at the time of completion of their respective treatments (16 weeks for 4‐MRG vs. 24 weeks for 6‐MRG), this difference was not significant (*p* = 0.9054). These findings suggested that, while the short‐course regimen may show an early‐stage advantage in modulating a specific subset of CD8^+^ cells, both regimens ultimately lead to similar outcomes, supporting their potential equivalence in long‐term efficacy.

### 3.6 Note on Statistical Analysis

This study was designed as an exploratory analysis to identify potential associations that warrant further investigation in future validation studies. To maintain sensitivity and avoid overlooking potentially meaningful signals, we chose not to apply corrections for multiple comparisons. Certain correlations exhibited *p*‐values approaching the conventional threshold for strong significance, indicating trends that merit follow‐up but did not meet strict significance criteria. These findings should be regarded as hypothesis‐generating and preliminary. Rigorous validation in independent cohorts will be essential to assess the reliability and generalizability of these associations.

## 4 Discussion

Detecting T‐cell immunophenotypes in the serum of TB patients offers promise for identifying immune signatures specifically linked to TB. However, traditional experimental methods have hitherto failed to establish a correlation between serum cytokine levels and disease severity. Here, we have established associations between 124 T‐cell phenotypes and four disease severity indicators, providing clinicians with new insights into the immunological landscape of TB that may inform future diagnostic or treatment strategies.

Of the 14 T‐cell subsets with distinct proportions that were associated with clinical severity, six (42.8%) were found to express PD‐L1. This finding indicates that PD‐L1 expression may play an important role in disease progression. As an immune checkpoint, high PD‐L1 expression in TB inhibits T‐cell activation and proliferation, thereby weakening immunity and affecting disease progression. Recent studies have shown that long‐term chronic infections are often accompanied by the depletion of antigen‐specific T cells characterized by functional defects and upregulation of a variety of inhibitory receptors [16]. In patients with pulmonary TB, similar patterns of T‐cell dysfunction have been observed in granulomatous tissues, where overexpression of inhibitory molecules (e.g., PD‐1 and LAG‐3) may contribute to immune suppression [17, 18, 19]. Multiple investigations involving both human patients and animal subjects have directly implicated the PD‐1/PD‐L1 pathway in the immunopathogenesis of TB. For instance, one study demonstrated that the PD‐1/PD‐L1 pathway modulates antigen‐specific cytotoxicity against M1‐type macrophage targets, disrupting the body's ability to effectively combat MTB. Additionally, Suarez et al. demonstrated how this pathway can lead to the suppression of T‐cell responses, allowing MTB bacteria to persist and the disease to progress. An increased frequency of PD‐L1^+^ cells contributes to immunosuppression, facilitating disease progression. This association may aid clinicians in assessing patients for disease severity.

This study is the first to identify an association between CD45RO^−/+^CD27^+^ CD8^+^ T‐cell subsets and DILI, representing a significant advancement in understanding its immunopathogenesis. Notably, we also identified three IL‐21^+^ cell subsets that may contribute to this condition. These findings contrast with previous research showing that activation of the IL‐21/signal transducer and activator of transcription 3 signaling pathway could protect against concanavalin A‐induced acute liver injury in mice [20]. However, this discrepancy may reflect fundamental differences in the disease context between that experimental model and DILI triggered by anti‐TB treatment in patients. By identifying specific DILI‐associated T‐cell subsets, we may be able to develop new biomarkers for risk stratification of patients undergoing anti‐TB treatment. Additionally, our findings lay the groundwork for exploring IL‐21‐targeted therapies aimed at preventing or mitigating DILI, offering a promising avenue for future translational research.

Another notable subset, CD45RO^−^CD27^+^ CD8^+^ T cells, was negatively correlated with disease severity, suggesting that this population may play a protective role in the host response to TB. Because we sought to analyze changes in antigen‐experienced T cells in the context of TB infection, we chose CD45RO as a marker instead of CD45RA, which is typically expressed on naïve T cells [21]. CD27, a T‐cell costimulatory molecule, supports antigen‐specific expansion of naïve T cells, is essential for the primary CD4^+^ and CD8^+^ T‐cell responses to infection, and plays a crucial role in the generation of T‐cell memory [22]. CD45RO^−^CD27^+^ represents a subset of naïve or incompletely activated CD8^+^ T cells that are capable of efficiently recognizing and killing cells infected by MTB. A decline in this population may impair the body's ability to clear intracellular bacteria, allowing MTB to multiply extensively within host cells (e.g., macrophages) and facilitating its spread. These findings highlight the potential of this potent subset in immune defense and may provide an important theoretical basis for optimizing and evaluating new TB vaccine strategies.

Our study demonstrates that differential immune responses to treatment regimens, particularly in relation to T‐cell phenotypes, may help guide therapeutic decision‐making. A key finding is that patients exhibiting a high frequency of CD45RO^−^IFN‐γ^+^ CD8^+^ T cells at treatment week 16 may be optimal candidates for short‐course regimens. This inference is based on our observation that, among multiple immunophenotypes analyzed, only IFN‐γ–producing naïve T cells (CD45RO^−^IFN‐γ^+^) showed a statistically significant difference at the 16‐week treatment timepoint. Notably, this difference disappeared by the end of standard full‐course therapy, suggesting the observed effect is treatment‐duration dependent.

This study has several limitations. First, the small sample size and lack of multivariate analysis may limit the reliability of the findings and their applicability to large‐scale settings. Second, because the study was conducted within a specific region and time frame, the patient population may not represent the full spectrum of TB cases. Third, the analysis focused on a limited set of *Mycobacterium*‐related T‐cell subsets, potentially overlooking other crucial immune indicators.

Future research should address these issues. Looking ahead, clinical studies should expand the sample size and incorporate multicenter designs with multivariate analyses to improve the reliability of the results. Additionally, advanced technologies such as single‐cell sequencing and proteomics could uncover novel immune signatures and potential clinical indicators. Strengthening collaboration between basic and clinical researchers will be essential to accelerate the translation of research insights into improved diagnostic tools and treatment strategies, ultimately enhancing patient outcomes.

## 5 Conclusion

This study addresses the gap in understanding the association between immunophenotype characteristics, DILI, and the severity of TB. We identified 14 immunophenotypes linked to TB progression, including PD‐L1^+^ subsets that may serve as novel biomarkers for assessing disease severity. Notably, we provide the first evidence that IL‐21^+^ cell populations may modulate DILI risk during anti‐TB treatment. In addition, CD45RO^−^IFN‐γ^+^ CD8^+^ T cells emerged as a temporal biomarker for tailoring treatment duration.

This study provides a framework for precision TB management by integrating immunophenotypic dynamics with treatment response kinetics. Key translational strategies include incorporating inhibitory receptors (e.g., PD‐1) in hematological monitoring, reducing IL‐21 levels as a preventive hepatoprotective measure, and avoiding drugs with confirmed hepatotoxic potential. Additionally, the identification of CD45RO^−^IFN‐γ^+^ CD8^+^ T cells as a treatment‐responsive biomarker may support individualized treatment duration, ultimately improving patient outcomes.

## Author Contributions

Wei Sha and Ying Wang designed the research. Yifan He performed research and wrote the manuscript. Xubin Zheng and Zihan Dang analyzed data. Yingying Chen contributed important reagents. Xiaohui Hao, Yidian Liu, and Peng Wang collected data. All authors have read and agreed to the final version of the manuscript.

## Ethics Statement

This is a substudy of parent study NCT03561753, which was approved by the Ethics Committee of Shanghai Pulmonary Hospital.

## Consent

Written informed consent was obtained from all participants in the parent study, and the substudy protocol was approved under the same ethical framework, with data usage explicitly covered in the original consent documents.

## Conflicts of Interest

The authors declare no conflicts of interest.

## Supporting information


**Table S1.** Antituberculosis drug doses prescribed in this study.

## Data Availability

The datasets generated and analyzed during the current study are available from the corresponding author on request.

## References

[1] World Health Organization , Global Tuberculosis Report, 2023 (World Health Organization, 2023).

[2] G. Gunther , J. Heyckendorf , J. P. Zellweger , et al., “Defining Outcomes of Tuberculosis (Treatment): From the Past to the Future,” Respiration 100, no. 9 (2021): 843–852. 34058739 10.1159/000516392

[3] P. P. Phillips , C. M. Mendel , D. A. Burger , et al., “Limited Role of Culture Conversion for Decision‐Making in Individual Patient Care and for Advancing Novel Regimens to Confirmatory Clinical Trials,” BMC Medicine 14 (2016): 19. 26847437 10.1186/s12916-016-0565-y PMC4743210

[4] World Health Organization , “WHO Consolidated Guidelines on Tuberculosis,” in Module 3: Diagnosis (World Health Organization, 2025). 40388555

[5] J. P. Zellweger , G. Sotgiu , M. Corradi , and P. Durando , “The Diagnosis of Latent Tuberculosis Infection (LTBI): Currently Available Tests, Future Developments, and Perspectives to Eliminate Tuberculosis (TB),” Medical Laboratory 111, no. 3 (2020): 170–183. 10.23749/mdl.v111i3.9983 PMC7809945 32624559

[6] W. S. Lim , A. Avery , O. M. Kon , and M. Dedicoat , “Anti‐Tuberculosis Drug‐Induced Liver Injury,” BMJ (Clinical Research ed.) 383 (2023): e074866. 10.1136/bmj-2023-074866 37890885

[7] V. T. A. Thu , L. D. Dat , R. P. Jayanti , et al., “Advancing Personalized Medicine for Tuberculosis Through the Application of Immune Profiling,” Frontiers in Cellular and Infection Microbiology 13 (2023): 1108155. 36844400 10.3389/fcimb.2023.1108155 PMC9950414

[8] M. I. M. Ahmed , N. E. Ntinginya , G. Kibiki , et al., “Phenotypic Changes on Mycobacterium Tuberculosis‐Specific CD4 T Cells as Surrogate Markers for Tuberculosis Treatment Efficacy,” Frontiers in Immunology 9 (2018): 2247. 30323818 10.3389/fimmu.2018.02247 PMC6172348

[9] T. Adekambi , C. C. Ibegbu , S. Cagle , et al., “Biomarkers on Patient T Cells Diagnose Active Tuberculosis and Monitor Treatment Response,” Journal of Clinical Investigation 125, no. 5 (2015): 1827–1838. 25822019 10.1172/JCI77990 PMC4598074

[10] Y. K. Yong , H. Y. Tan , A. Saeidi , et al., “Immune Biomarkers for Diagnosis and Treatment Monitoring of Tuberculosis: Current Developments and Future Prospects,” Frontiers in Microbiology 10 (2019): 2789. 31921004 10.3389/fmicb.2019.02789 PMC6930807

[11] X. Zheng , X. Gui , L. Yao , et al., “Efficacy and Safety of an Innovative Short‐Course Regimen Containing Clofazimine for Treatment of Drug‐Susceptible Tuberculosis: A Clinical Trial,” Emerging Microbes & Infections 12, no. 1 (2023): 2187247. 36872899 10.1080/22221751.2023.2187247 PMC10026740

[12] Y. Xia , H. Chen , C. Zhang , Y. Zhao , J. Cheng , and H. Zhang , “Guidelines for the Prevention and Control of Tuberculosis in Schools: Recommendations From China CDC,” China CDC Weekly 3, no. 2 (2021): 34–38. 34594902 10.46234/ccdcw2021.009 PMC8392894

[13] World Health Organization , WHO Consolidated Guidelines on Tuberculosis: Module 4: Treatment ‐ Drug‐Susceptible Tuberculosis Treatment [Internet] (World Health Organization, 2022). 35727905

[14] Council for International Organizations of Medical Sciences (CIOMS) , Drug‐induced liver injury (DILI): Current Status and Future Directions Fordrug Development and the Post‐Market Setting (Council for International Organizations of Medical Sciences, 2020).

[15] B. O. Schroeder and F. Backhed , “Signals From the Gut Microbiota to Distant Organs in Physiology and Disease,” Nature Medicine 22, no. 10 (2016): 1079–1089. 10.1038/nm.4185 27711063

[16] S. Ando and K. Araki , “CD8 T‐Cell Heterogeneity During T‐Cell Exhaustion and PD‐1‐Targeted Immunotherapy,” International Immunology 34, no. 11 (2022): 571–577. 35901837 10.1093/intimm/dxac038 PMC9533227

[17] J. Li , C. Jin , C. Wu , and J. Huang , “PD‐1 Modulating Mycobacterium Tuberculosis‐Specific Polarized Effector Memory T Cells Response in Tuberculosis Pleurisy,” Journal of Leukocyte Biology 106, no. 3 (2019): 733–747. 30861206 10.1002/JLB.MA1118-450RR

[18] C. L. Day , D. A. Abrahams , R. Bunjun , et al., “PD‐1 Expression on Mycobacterium Tuberculosis‐Specific CD4 T Cells Is Associated With Bacterial Load in Human Tuberculosis,” Frontiers in Immunology 9 (2018): 1995. 30233588 10.3389/fimmu.2018.01995 PMC6127207

[19] A. Singh , A. Mohan , A. B. Dey , and D. K. Mitra , “Inhibiting the Programmed Death 1 Pathway Rescues *Mycobacterium tuberculosis*‐Specific Interferon Gamma‐Producing T Cells From Apoptosis in Patients With Pulmonary Tuberculosis,” Journal of Infectious Diseases 208, no. 4 (2013): 603–615. 23661793 10.1093/infdis/jit206

[20] J. Ahodantin , J. Wu , M. Funaki , et al., “Siglec‐H(−/−) Plasmacytoid Dendritic Cells Protect Against Acute Liver Injury by Suppressing IFN‐Gamma/Th1 Response and Promoting IL‐21(+) CD4 T Cells,” Cellular and Molecular Gastroenterology and Hepatology 18, no. 3 (2024): 101367. 38849082 10.1016/j.jcmgh.2024.101367 PMC11296256

[21] M. G. T. Ahmed , A. Limmer , and M. Hartmann , “CD45RA and CD45RO Are Regulated in a Cell‐Type Specific Manner in Inflammation and Sepsis,” Cells 12, no. 14 (2023). 10.3390/cells12141873 PMC10378241 37508538

[22] J. Hendriks , L. A. Gravestein , K. Tesselaar , R. A. van Lier , T. N. Schumacher , and J. Borst , “CD27 Is Required for Generation and Long‐Term Maintenance of T Cell Immunity,” Nature Immunology 1, no. 5 (2000): 433–440. 11062504 10.1038/80877

