# Supplementary material for: Association of T‐Cell Profiles With Disease Severity, Drug‐Induced Liver Injury, and Treatment Completion in Tuberculosis
Source: Clin Respir J. 2025 Aug 1;19(8):e70114. doi: 10.1111/crj.70114 (PMC12314307; doi:10.1111/crj.70114)
Supplement: Supplementary file 1 — Table S1. Antituberculosis drug doses prescribed in this study. [file CRJ-19-e70114-s001.docx]

**Supplement**

**Inclusion and exclusion criteria**

Inclusion criteria were mainly the followings:

1. Newly diagnosed and untreated bacteriologically-confirmed tuberculosis (TB) patient

2. Pulmonary lesion consistent with TB by radiological examination

3. Positive sputum culture, identification of bacterial type confirmed *Mycobacterium tuberculosis*. MGIT drug sensitivity test (DST) results are sensitive of the first-line drugs (isoniazid, streptomycin, rifampicin and ethambutol).

4. Age 18-65 years old

5. Males or non-pregnant, non-nursing females

6. Women of child-bearing potential who are not surgically sterilized must agree to practice a barrier method of contraception or abstain from heterosexual intercourse during study drug treatment.

7. Laboratory parameters done at or within 14 days prior to screening:

a. Serum or plasma aminotransferases (AST, ALT) less than 3 times the upper limit of normal

b. Serum or plasma total bilirubin less than or equal to 2.5 times the upper limit of normal

c. Serum or plasma creatinine level less than or equal to 2 times the upper limit of normal

d. Serum or plasma potassium level greater than or equal to 3.5 meq/L

e. Haemoglobin level of 7.0 g/dL or greater

f. Platelet count of 100,000/mm^3^ or greater

g. For women of childbearing potential, a negative pregnancy test is

required during screening

8. Provides written informed consent

9. Willingness and ability to attend scheduled follow-up visits and undergo study assessments.

Exclusion criteria were mainly the followings:

1. Unable to take oral medications

2. History of allergy or intolerance to any of the study drugs

3. Serum aminotransferase (AST or ALT) 3x upper limit of normal or higher

4. Pregnant or nursing females, or plan to become pregnant or nurse during the study period

5. Males planning to conceive a child during the study or within 6 months of cessation of treatment.

6. Any treatment directed against active TB within 6 months preceding initiation of study drugs.

7. Suspected or documented TB involving the central nervous system and/or bones and/or joints, and/or miliary TB and/or pericardial TB.

8. HIV infected

9. HBV infected or HCV infected (these increase the risk of TB-drug induced hepatotoxicity)

10. Individuals will be excluded from enrolment if, at the time of enrolment, their *M. tuberculosis* isolate is already known to be resistant to any of the study drugs.

11. COPD and its comorbidities.

12. Other chronic disease: Diabetes mellitus type 2, chronic kidney disease and rheumatoid arthritis.

13. Other medical conditions, that, in the investigator's judgment, make study participation not in the individual's best interest.

14. Current or planned incarceration or other involuntary detention

15. Having participated in other clinical studies with dosing of investigational agents within 8 weeks prior to trial start or currently enrolled in an investigational study that includes treatment with medicinal agents. Subjects who are participating in observational studies or who are in a follow up period of a trial that included drug therapy may be considered for inclusion.

Table S1 Anti-tuberculosis drug doses prescribed in this study

| Drug | Weight-banded daily dose (gram) | | Frequency (times/day) |
| --- | --- | --- | --- |
|  | Weight < 50 kg | Weight ≥ 50 kg |  |
| Rifampicin | 0.45 | 0.6 | 1 |
| Isoniazid | 0.3 | 0.3 | 1 |
| Pyrazinamide | 1.5 | 1.5 | 1 or 3 |
| Ethambutol | 0.75 | 1.0 | 1 or 2 |
| Clofazimine | 0.15 | 0.15 | 1 or 3 |
| Prothionamide | 0.6 | 0.6 | 3 |

**Microbiological work**

Bacterial culture was performed via a mycobacterial growth indicator tube (MGIT) using the BACTEC MGIT 960 system (Becton Dickinson, Franklin Lakes, NJ, USA) in the quality-controlled TB reference laboratory of the Shanghai Pulmonary Hospital. Positive *M. tuberculosis* strains at baseline were tested for susceptibility to 12 drugs, including rifampicin, isoniazid and ethambutol using a Sensititre MYCOTB MIC plate (Trek Diagnostic Systems, Cleveland, OH, USA). Sixteen-hour pooled sputum collections (from 4 pm to 8 am) were used for evaluation of early bactericidal activity (EBA) by enumeration of colony forming units (CFU). Samples were collected before treatment (day 0) to establish baseline counts of CFU, and at 2, 4, 7, 10, 12 and 14 days after treatment initiation. Pooled specimens were collected in sterile disposable 50-ml polypropylene centrifuge tubes at each sampling point and stored at 2 to 8°C prior to processing. In brief, specimens were homogenized by adding 10% dithiothreitol to reach a final concentration of 1% and agitated on a platform shaker for 20 min. Then, a series of 10-fold dilutions using sterile Tween-80/0.85% saline was prepared (10^−1^, 10^−2^, 10^−3^, 10^−4^, 10^−5^ and 10^−6^) and inoculated in duplicate on Middlebrook 7H10 agar plates enriched with oleic acid-citrate-dextrose (BD Biosciences, NJ, USA). After 3 to 4 weeks of incubation at 37°C, CFU were enumerated at the dilution that yielded 20 to 200 visible colonies.
